# An ETP model (exclusion-tolerance-progression) for multi drug resistance

**DOI:** 10.1186/1742-4682-2-17

**Published:** 2005-04-27

**Authors:** Subburaj Kannan

**Affiliations:** 1 Division of Gastroenterology, School of Medicine, University of Pennsylvania, Philadelphia, PA, 19104, USA

**Keywords:** ETP Model, Drug resistance, Drug sensitivity, Physiological drug resistance, Pathological drug resistance

## Abstract

**Background:**

It is known that sensitivity or resistance of tumor cells to a given chemotherapeutic agent is an acquired characteristic(s), depending on the heterogeneity of the tumor mass subjected to the treatment. The clinical success of a chemotherapeutic regimen depends on the ratio of sensitive to resistant cell populations.

**Results:**

Based on findings from clinical and experimental studies, a unifying model is proposed to delineate the potential mechanism by which tumor cells progress towards multi drug resistance, resulting in failure of chemotherapy.

**Conclusion:**

It is suggested that the evolution of multi drug resistance is a developmentally orchestrated event. Identifying stage-specific time windows during this process would help to identify valid therapeutic targets for the effective elimination of malignancy.

## Background

The phenomenon of drug resistance is a distinct and multifactorial entity culminating in the failure of therapeutic regimens in clinical oncology. From the clinical perspective, the emergence of drug resistance is determined by the rate of tumor growth, in conjunction with the remission index subsequent to chemotherapy. In contrast, experimental model(s) for studying drug resistance involve either homogeneous cell populations or co-culture models where the time frame ranges from a few days to a week at the most. It is obvious that the biochemical and collective physiological process that coexist in the cancer patient are totally distinct and do not warrant direct comparison with experimental data.

A tumor mass encompassing approximately 10^11 ^to 10^12 ^cells is considered a lethal tumor burden. Depending on the sensitivity of a given tumor cell population to anti-neoplastic drug(s) (chemotherapeutic agents), and the dose administered, the effectiveness of the therapy, referred to as "Cell Kill", is determined. "Cell Kill" depends on the inherent susceptibility of the tumor burden. The "Cell Kill" of a given tumor burden varies between 90 % to 99.99%. If we assume that, under the best therapeutic regimen, one round of chemotherapy or radiation therapy would be likely to achieve a 99.99% "cell kill" in a tumor burden of 10^11^cells, this would reduce the tumor burden to 10^8 ^cells. It should be noted that chemotherapy and radiation therapy exert considerable toxic effects on normal cells; because of this factor, the treatment regimen is staged in cycles. It is reasonable to presume that both the normal cells and the less sensitive tumor cells would be likely to proliferate with one or more defects after each cycle of the therapy.

Even if the most fortunate circumstances were to prevail for early detection followed by treatment, the size of the "tumor burden", the sensitivity of the tumor cells and the effectiveness of the therapy ("Cell kill") remain decisive in determining the outcome of the therapy. Clinical observation shows that relapse or recurrence of the tumor is always a possibility. This is partly due to the fact that diagnostic procedures are inadequate for detecting as few as 10^6 ^or 10^8 ^tumor cells in cancer patients. This technical shortcoming confers a growth advantage on both undetectable and insensitive tumor cells. Under these circumstances, should there be a relapse, the tumor burden would probably be composed of a heterogeneous population of tumor cells. These include both drug sensitive and drug resistant cells in either proliferating or dormant states. According to Gompertzian Kinetics, as the tumor burden increases, the number of proliferating cells would decrease. Also, it is known that in a given tumor burden, a considerable number of cells are in the resting phase; these are not sensitive to chemotherapy or radiation therapy [1].

As a result it would require a much higher dose of chemotherapeutic drugs or radiation therapy to achieve the maximal "Cell Kill" in a given tumor burden. It is known that higher drug doses are often correlated with increased response rates in terms of the effective and maximal "Cell Kill", thus offering a window of opportunity for cure (complete remission). However, there is no assurance that complete remission would be the immediate outcome after a given therapeutic regimen. It is known that the effectiveness of an anti-neoplastic drug depends on **i) **the half life of the drug ***in vivo***, **ii) **the rate and **iii) **amount of the drug being absorbed (bioavailability), and **iv) **the toxicities of biologically active metabolites of the drug [2-4].

The biologically active form of a drug, and the effective range of radiation therapy, are not uniformly distributed, so the entire tumor burden is not reached. Therefore, in a given tumor burden, the entire cell population is not exposed to effective therapy, leaving a finite region insufficiently exposed to the drug or its biologically active metabolite(s). Considering these factors in the context of ongoing therapy, it is common practice to re-evaluate patients after 2 or 3 cycles of chemotherapy to determine its effectiveness. Depending on the toxicity profile and the rate of tumor progression versus "Cell Kill", either the therapy is continued or multiple drugs are used to achieve maximal "Cell Kill" to obtain complete remission.

Here, I advance a hypothesis for multi drug resistance based on the aforementioned factors: the ETP model. The founding factors are: **1. **The sigmoidal curve depicting the toxicity-dose relationship for a given chemotherapeutic drug indicates that lower doses give lower toxicity but less "Cell Kill", whereas an increased dose would increase the toxicity with a better "Cell Kill" [5,6]. However the optimal dose required for a given therapeutic compound to achieve a maximal "Cell Kill" for a particular malignancy with minimum cytotoxicity is not well defined. This paves the way for cells within the tumor burden to acquire and evolve one or more mechanism(s) for survival under the drug-induced toxic environment during and after treatment. Therefore, it follows that the surviving tumor cells in a given "tumor burden" should be viewed as the best-suited or best-adapted for withstanding these toxic effects as a result of acquiring a relevant genotype and/or phenotype conferring "multi drug resistance" [7,8].

**Figure 1:**
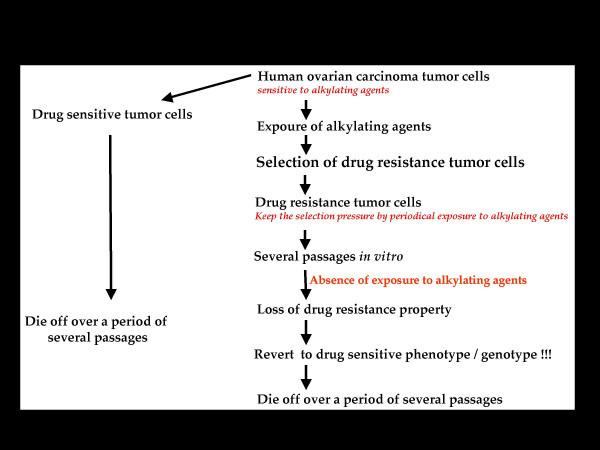
Schematic representation of an experimental drug resistance model.

**2. **In terms of experimental data, I have consistently observed that periodic exposure to alkylating agents is a principal requirement for retaining the drug-resistance property of the drug-resistant variant of human ovarian carcinoma compared to the drug-sensitive tumor cells ***in vitro***. If no drug treatment were provided, would the drug-resistant variant revert to a sensitive phenotype and subsequently die? Absence of drug exposure of resistant cells indeed causes reversion to a drug sensitive phenotype, which is intriguing as it implies that drug resistance is a transient phenomenon not an everlasting property, at least *in vitro*. Also, as the selection pressure is maintained with periodic drug treatment, the doubling rate of the drug resistant cells decreases compared to the drug-sensitive phenotype (Figure 1). Obviously, a considerable difference in biochemical properties between these two cell lines is to be expected; however, this is not the case (**Kannan**, **Unpublished observations**). On the contrary, if chemotherapeutic or radiation therapy is discontinued, relapse with or without an aggressive increase in tumor burden is observed in a cancer patient.

Absence of therapy does not result in a complete remission paralleling the complete reversion to drug sensitivity that is observed ***in vitro***. The phenomenon is rare, indeed scarcely possible, in the clinical scenario. As such, there is no direct correlation between clinical case reports and experimental data. This contradiction warrants critical appraisal of the published literature, which is seminal and has exerted a profound impact on our understanding of the most complex and still unattainable goal of complete recovery in any given cancer treatment. Based on extensive analysis of the published literature and experimental evidence, I propose a model to account for the incongruity or discrepancy between the experimental and clinical drug resistance data.

Although the biochemical basis of drug resistance has been extensively studied in both in vitro model systems and clinical specimens, no correlation has been forthcoming. Considering the disparity between experimental and clinical findings, it is clear that we currently lack an understanding of the physiological basis of the evolution of drug-resistant cells. The expression of proteins that play a pivotal role in cellular drug resistance, such as P-glycoprotein, MRP-1 and other members of the ATP-binding cassette family of efflux protein(s), has been shown to decline at the time of relapse compared to the initial presentation of a given type of cancer. However, this observation has not been corroborated in all types of cancer. MRP-1 mediated drug efflux has been shown to correlate with an elevated level of intracellular glutathione (GSH), GSH synthesis or glutathione S-transferase (GST) activity. GST-mediated glutathionyl S-conjugates are known to be removed from the intracellular milieu by an energy-dependent process, similar to that seen with MRP-1, which is mediated by ATP-binding cassette proteins. It is known that GST is expressed in four different isoforms (α, , π, ) but it has not yet been confirmed that expression of GST-π confers drug resistance or whether this isoform is present in drug-resistant cells [9].

Among the properties that differentiate tumor cells from normal cells are the presence of growth factors potentiating vascular growth, highly heterogeneous oxygen tension distributions, extreme acidic or alkaline pH, higher rates of glucose delivery and utilization, and finally, a state of hypoxia with an acidotic environment that is noncycling. This, in turn, endows the cell population with a lack of uniform sensitivity to different families of chemotherapeutic drugs. Oxidative stress has been demonstrated to induce genomic instability at a much higher rate than is seen in drug-sensitive cells. All these factors contribute to the development of drug resistance [10].

In addition, drug-resistant cells have evolved mechanisms for bypassing apoptosis (the controlled form of cell death due to dehydration, shrinkage, and fragmentation of the nucleus, eventually leading to phagocytosis by macrophages) and necrosis (a traumatic but passive form of cell death due to the dysfunction of ion-transporting proteins, cell swelling and lysis and associated with the release of inflammatory mediators) [11].

## Rationale

**Figure 2:**
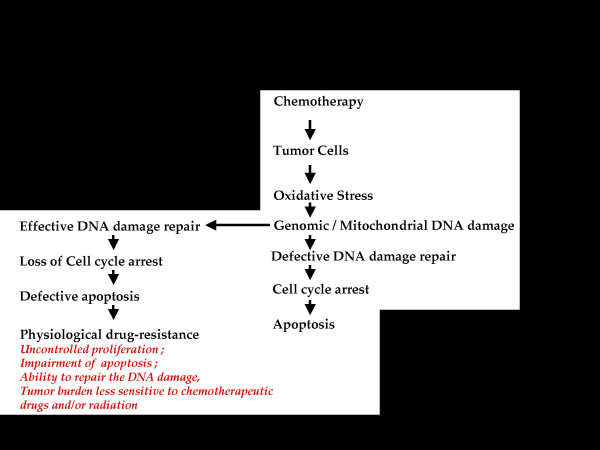
**Phase I: **Evolution of physiologically drug resistant cells by "exclusion" from chemotherapy-sensitive tumor cells.

**Figure 3:**
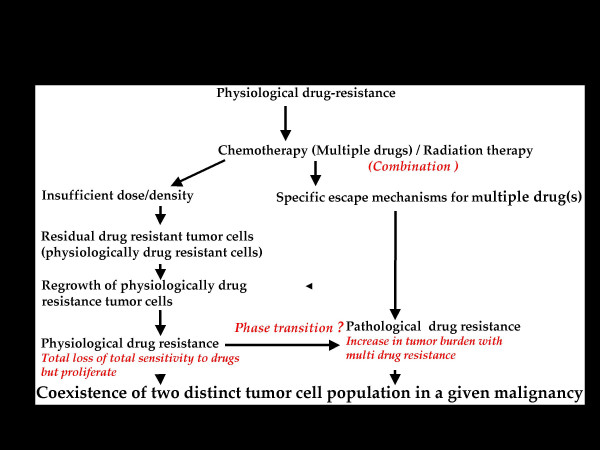
**Phase II: **Existence of multiple mechanism(s) in a tumor burden for efficient evolution of pathologically drug resistant cells and "toleration" of coexisting physiologically drug resistant tumor cells.

**Figure 4:**
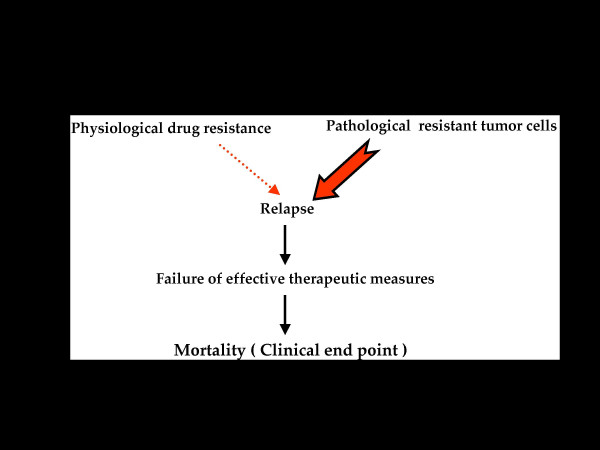
**Phase III: **Progression of pathologically drug resistant tumor cells leading to mortality.

Therefore, it is hypothesized that the formation of drug-resistant tumor cells occurs in at least two distinct stages, namely physiological drug resistance and pathological drug resistance. Physiological drug resistance denotes the stage during which the cells are afflicted with a variety of cellular stress signals and become more susceptible to the type of damage likely to be inflicted by chemotherapeutic drugs. Physiological drug resistance is characterized by uncontrolled proliferation; impairment of apoptosis; ability to repair DNA damage; and increasingly lower sensitivity to chemotherapeutic drugs and/or radiation. Thus, cells in this group are distinct from normal cells. Subsequent to chemotherapy, depending on its effectiveness, multi drug resistance tumor cells evolve within the tumor burden. After several courses of therapy, due to several contributing factors, the host is overwhelmed with predominantly multi drug-resistant cells (referred to as pathologically drug resistant tumor cells) which, in turn, confer unresponsiveness to chemotherapeutic agents and/or adjuvant therapeutic treatment, leading to mortality. Figures 2, 3 and 4 summarize the hypothesis advanced here; they depict the scheme of events following chemotherapeutic treatment and the significance of the two distinct tumor cell populations in leading to the failure of a therapeutic regimen.

## Hypothesis

### Phase I. *Exclusion*

A fundamental characteristic of malignancy is genetic instability, which leads to a heterogeneous cell population endowed with altered morphologies, invasiveness, drug resistance and neovascularization, properties acquired through genetic mutation and aberrant gene expression. In addition, chemosensitivity is most likely caused by genetic changes; it is an acquired feature that appears at one specific stage and may be lost as the tumor burden progresses. It is hypothesized that tumor cells progress through two distinct stages, namely 'physiological drug resistance' and 'pathological drug resistance'. Subsequent to chemotherapy, the tumor cells are in a state in which drug sensitivity is similar to that in the corresponding normal cells. Within the tumor burden, depending on their drug sensitivity, a more limited number of tumor cells than corresponding normal cells are damaged by chemotherapeutic agents.

As shown in Figure 2, following chemotherapy, the cells are subjected to oxidative stress, which has been shown to induce and sustain genomic and mitochondrial DNA damage. Notwithstanding the mechanisms for repairing such DNA damage, the loss of genomic DNA integrity, entailing the down-regulation of apoptotic suppressor proteins, drug-resistance suppressor proteins or cell cycle regulatory proteins, obviously programs the cells to undergo cell cycle arrest, culminating in apoptosis.

Tumor cells capable of repairing DNA damage but retaining the upregulation of cell cycle regulatory proteins and drug-resistance proteins are likely to survive both oxidative stress and apoptosis [12,13]. It is speculated that this defined sub-population is likely to represent the initially drug-resistant cells, where the phenomenon is referred as **"physiological drug resistance"**.

It is also possible that a select population of tumor cells may retain drug resistance and increase the expression of pro-apoptotic genes while losing the cell cycle regulatory protein(s). Such cells would be likely to undergo apoptosis. Thus, the evolving tumor would most likely contain a defined population of cells tolerant of oxidative stress and toxic drug effects, and also capable of bypassing apoptosis. *Therefore, this initial phase is an exclusion phase during which tumor cells that have lost cell cycle control and tumor suppressor proteins are excluded from the rest of the tumor burden as an evolving population endowed with physiological drug resistance*.

However, this selection process does not ensure that the entire physiologically drug resistant fraction of the tumor burden has become resistant to all drugs (Figure 2). To support my argument that two distinct form of tumor cells progress and lead to drug resistance mediated failure of therapy, I invoke the process of "apomixis" [14] to erect a hypothesis that depends on the presence of *two hypothetical types of cell *in the same somatic tissue (tumor burden) and a potential exchange of genetic material between them. "Apomixis" is a process that accounts for ***asexual***reproduction in higher forms of life where sexual reproduction is the norm. In essence, the successful evolution of pathologically drug-resistant cells may or may not follow a pattern. The foremost factors determining the success of this process are cellular genetic defects (mutations), and the amount of chemotherapy and/or radiation the patient will subsequently undergo. Together, these factors determine or influence either the physiologically or the pathologically drug resistant cells. In successive generations, resistant cells are likely to adopt at least one additional molecular mechanism for mounting an effective defense against adjuvant therapy, after the initial radiation or chemotherapy has failed, plausibly because of **apomixis**.

### Phase II. *Tolerance*

Tumor cells that are not susceptible to the toxic effects of chemotherapeutic drugs and are tolerant of oxidative stress are expected to possess one or more molecular mechanisms to protect and maintain the proteins essential for survival. Such tumor cell populations show physiological drug resistance. The sustained cytotoxic effects of chemotherapeutic drugs would be likely neither to induce genomic instability nor to affect cell cycle progression in such a population. Also, insufficient exposure to the therapy would leave a defined fraction of the tumor cells to re-grow with the property of physiological drug resistance and remain in the tumor burden. In addition to these physiological possibilities, it seems reasonable to propose that expression of several cell cycle regulatory proteins will be lost and that cell cycle arrest will become dissociated from DNA damage. In turn, accumulated DNA damage and uncontrolled cell cycle progression with impaired apoptotic pathways will confer increased resistance to chemotherapeutic drugs and/or adjuvant therapeutic treatment, protecting against cell death and sustaining tumor cell proliferation.

Therefore, this subpopulation of tumor cells is selected to progress towards pathological drug resistance with or without a specific escape mechanism for multiple drugs (e.g. increased glutathione levels and altered DNA repair, loss of cell cycle check point kinases). Also, it suggested that pathological drug resistance probably represents a stage during which resistance to various cytotoxic insults increases markedly. It is also speculated that these distinct tumor cell populations would probably coexist in a tumor burden by "tolerating" each other (Figure 3).

A mechanistic working hypothesis is presented in Figure 3, in which physiological drug resistance progresses to increased insensitivity to multiple drugs and subsequently to multi drug resistance. With subsequent tumor progression, several defense mechanisms may be lost, including dissociation of cell cycle arrest from DNA damage. Further, clonal progression of the 'pathologically drug resistant' cells may emerge as a result of drug-specific escape mechanisms and the impairment of both triggering and effectors mechanisms of apoptosis. Essentially, failure of all options in a clinical chemotherapeutic regimen produces a shift from physiological drug resistance to pathological drug resistance. Advanced clinical stages, representing the failure of multiple episodes of a therapeutic regimen, would be more likely to contain pathologically drug-resistant cells that are beyond the chemosensitive window.

Overall, it is still a strong possibility that both physiologically and pathologically drug-resistant cells, together with yet unknown drug-sensitive cells, might coexist in a tumor burden, complicating any viable alternative approach to therapy. The promise of any therapeutic measure at this point would largely depend on the properties of the predominantly surviving cell population in the tumor burden. Regrettably, this would mean that the cancer patient is losing ground in therapy and reaching the clinical endpoint, which is not yet conclusive at this point of the treatment.

*Do the pathologically drug resistant tumor cells secrete some unidentified factor(s) or adopt a novel mechanism(s) to transform physiologically drug resistant tumor cells to pathologically drug resistant ones? It has been suggested that such a **"phase transition" **is a strong possibility *[15].

### Phase III. *Progression*

As shown in Figure 4, should the pathologically drug resistant tumor cells overwhelm the tumor burden, with or without the concomitant presence of physiologically drug resistant cells, it is likely that the relapse may lead towards complete failure of any remaining therapy. Relapses in cases of metastatic tumor burdens cause deterioration of the clinical scenario; metastatic tumors are more aggressive, in particular with a pathologically drug-resistant tumor burden. Progression of pathologically drug-resistant cells would most likely occur because of clonal dominance under the selection pressure imparted by the chemotherapy. In summary, a tumor burden that already contains more pathologically resistant cells would make the most intensive therapeutic regimen a futile exercise. since the pathologically resistant tumor cells would be insensitive as well as resistant to all forms of therapy. At this point it is reasonable to conclude that the cancer patient has reached the end point, meaning mortality.

## Conclusion

In this hypothesis, I have considered the multiple mechanism(s) of selection and proliferation in a distinct tumor cell population, namely pathologically drug resistant tumor cells, in the tumor burden, leading to the total failure of chemotherapy or an adjuvant therapeutic regimen. Furthermore, the following four characteristic properties of tumor cells may determine the pattern of drug resistance: 1. Absence of contact inhibition/uncontrolled proliferation; 2. Absence of apoptotic/necrotic mechanisms; 3. Multifactorial (epigenetic) up-regulation of drug resistance genes; 4. Sustained oxidative stress-mediated dysregulation of metabolic pathways. All the aforesaid factors would be likely to play pivotal roles in a developmental stage-specific manner, but not all at once. Delineating the specific molecular determinants conferring physiological versus pathological drug resistance genotype/phenotypes would be essential for providing an effective measure to attenuate the impact of multi drug resistance and clinical failure of the current therapeutic regimen.

## Author's contributions

SK generated the hypothetical scheme and formulated the hypothesis after a careful review of the appropriate literature.

The author does not have any competing financial or intellectual property interests with any party or association with members of the pharmaceutical manufacturing industry. The author declares that nearly all of the sequences, and the methods for generating the diadenosine polyphosphate hydrolases (Hint, Fhit, and GalT) (Biochemistry. 2002 41(29):9003-14.) protein family, purine-/pyrimidine-receptor sequences and the ectonucleotidase protein family, are in the public domain as of April 25, 2005. Therefore, the modified reagents and/or probe(s) originating from published sequences of the aforesaid protein family in the context of the scheme proposed in this article are part of one or more manufacturer(s) which are part of the impending U.S. or International patent application(s).
